# Development of the Team Evaluation and Assessment Measure Quality Improvement (TEAM‐QI) and Proof‐Of‐Concept Testing in Maternity Teams

**DOI:** 10.1111/nhs.70049

**Published:** 2025-02-03

**Authors:** Sarah Beck, Jenny Harris, James Green, Benjamin W. Lamb, Mehrnoosh Aref‐Adib, Debra Bick, Cath Taylor

**Affiliations:** ^1^ Faculty of Health and Medical Sciences, School of Health Sciences University of Surrey Surrey UK; ^2^ Department of Urology Whipps Cross Hospital, Barts Health NHS Trust London UK; ^3^ Department of Uro‐Oncology University College London Hospitals NHS Foundation Trust London UK; ^4^ Department of Obstetrics and Gynaecology Whipps Cross University Hospital, Barts Health NHS Trust London UK; ^5^ Warwick Clinical Trials Unit, Warwick Medical School University of Warwick Warwick UK

**Keywords:** intervention, maternity, multidisciplinary team, quality improvement, teamwork

## Abstract

Poor teamwork is often implicated in serious healthcare delivery failings, leading to calls for effective team improvement interventions. Taking a complex, adaptive systems perspective, we adapted an oncology team quality improvement program to make it appropriate for other areas of clinical care. Study phases included: (1) meetings with National Health Service, policy and service user representatives (*n* = 19), a rapid review of existing maternity teamwork interventions, and mapping of the proposed program content to an evidence‐based model of team effectiveness; (2) feasibility and acceptability testing of the team questionnaire component, and content analysis of free‐text responses with four maternity teams within two NHS Trusts (*n* = 26). Meetings with representatives highlighted the importance of non‐punitive, continuous team‐led assessment, and the ability to compare performance to similar teams while enabling adaptability to different team types. Program content mapped well to known components of team effectiveness. Internal consistency of the questionnaire was acceptable (Cronbach alpha = 0.79–0.92). Most team members (76.9%) reported benefits in identifying priorities for improvement. Preliminary proof of concept was supported but larger‐scale evaluation including testing in other clinical areas is warranted.

**Summary**

- Healthcare teamwork interventions typically focus on team dynamics and cultures, or the safety of performing high risk procedures. Few consider the systems‐wide factors that impact on day‐to‐day team effectiveness.
- We describe the development of an evidence‐based quality improvement programme co‐designed to help healthcare teams identify and address the micro, meso and macro barriers to effective teamworking.
- Initial results demonstrate the programme maps on to components known to influence optimal teamwork. Findings support the acceptability and utility of the programme among team members within maternity settings. Further testing of the programme is now warranted.

## 1 Introduction

Effective teamwork is essential for high‐quality health care (Manser 2009; Mazurenko et al. 2016). However, its success depends on effective collaboration between multiple, diverse and changing individuals, disciplines and allied health services (West and Lyubovnikova 2013; Schmutz, Meier, and Manser 2019), all within a complex environment with daily challenges and conflicting demands (Ramanujam and Rousseau 2006). Effective teams enhance patient safety, clinical performance, and standards of care (Manser 2009; Schmutz and Manser 2013). Additionally, effective teamworking contributes to retaining a skilled workforce (Schmutz, Meier, and Manser 2019; LePine et al. 2008). Globally, healthcare organizations emphasize the importance of effective teamwork, but there is evidence of wide variability in team performance and effectiveness (Australian Commission for Safety and Quality in Health Care 2020; Lamb et al. 2011; National Maternity Review 2016). For example, in England's 2023 National Health Service (NHS) Staff Survey (NHS Staff Survey 2022), only 51.4% of respondents agreed that teams within their organization collaborated effectively to achieve their objectives, and 48.7% expressed confidence in their organization's ability to address their concerns. Furthermore, prominent reports, particularly those based in maternity and neonatal services, have emphasized the impact of dysfunctional team dynamics and cultures (Australian Commission for Safety and Quality in Health Care 2020; Francis 2013; Kirkup 2022). These reports highlight the risks to patient care, including adverse outcomes such as avoidable death, caused by poor communication, complacency, and a lack of accountability within teams. Consequently, in the UK the government has acknowledged the role of improving teamwork to improve patient safety (Department of Health and Social Care 2023) and the three‐year delivery plan highlights the importance of supporting staff with professionalism, creating a culture of safety in organizations, and training together as a team (NHS England 2023). Taken together, this context has led to a growing emphasis on utilizing teamwork interventions to foster cultures of effective teamwork (Zajac et al. 2021). These interventions have been broadly described as tools, training, and quality improvement programs (definitions provided in Table S1). To date, much attention in teamwork research has focused on tools and training to improve the safety of specific procedures (e.g., surgery) or events (e.g., conduct of team meetings). Some widely adopted examples include the Team Climate Inventory (TCI) which has been widely applied within healthcare settings and designed to assess vision, participative safety, task orientation, and support for innovation (Anderson and West 1998), and Team Strategies and Tools to Enhance Performance and Patient Safety (TeamSTEPPS) which provides an evidence‐based framework and suite of tools and techniques to improve patient safety through enhanced communication and teamwork skills (King et al. 2008). Health care is an example of a complex adaptive system, involving numerous interacting features including patients, healthcare professionals, organizations, technology, and regulatory frameworks (Greenhalgh and Papoutsi 2018). However, few interventions consider the complex team functions across a whole patient pathway. For example, how different specialities support a cancer patients care from diagnosis through to follow‐up, discharge or into palliative care. Or in maternity services how service users, with varying levels of risk, are supported from the first scan to the end of pregnancy or early postpartum period, combined with the interpersonal team member and the organizational/resource influences on effectiveness (Harris et al. 2022).

More generally, the COVID‐19 pandemic has expedited changes in working practices, leading to a greater reliance on virtual ways of working, with team members communicating and convening online as “virtual teams.” While this has benefits (e.g., increased flexibility, easier access to patient information, and reduced travel time), it also provides challenges, including potential negative impacts on communication and interpersonal relationships in teams (Groothuizen et al. 2023). Due to the rapid pace of change, however, teamwork interventions that encompass these changes to working are lacking.

Previously, an evidence‐based assessment and feedback program specifically focused on improving teamwork in oncology (MDT‐FIT (Multi‐Disciplinary Team Feedback for Improving Teamworking)) was developed with more than 100 cancer multidisciplinary teams (MDT's) (Taylor et al. 2021; National Cancer Action Team 2010). Its component tools were validated (Taylor et al. 2012; Harris et al. 2016), and the program was evaluated as acceptable, feasible to implement and beneficial to team improvement (Taylor et al. 2021). The MDT‐FIT program comprised a questionnaire measure completed anonymously by each team member (Team Evaluation and Assessment Measure, TEAM) (Taylor et al. 2012) and an external observer assessment (Meeting Observational Tool, MDT‐MOT) (Harris et al. 2016), both of which were automatically synthesized into a feedback report sent to all team members via an online platform for discussion in a facilitated meeting where actions for improvement were agreed (Taylor et al. 2021). MDT‐FIT was codesigned with healthcare teams to be owned by the team and its leadership; for example, they decide on its timing and only the team members and their meeting facilitator have access to the feedback report. Additionally, it was codesigned to be run using observers and facilitators independent to the team, but from within the same organization (typically respected senior clinicians or managers) to support shared learning and implementation of the teams' action plan within the organization (Taylor et al. 2021).

Against the backdrop of evidence of serious failures in teamwork leading to instances of poor and dangerous care in areas of healthcare beyond oncology (Francis 2013; Kirkup 2022; Ockenden 2022), we, as the developers of MDT‐FIT (CT, JH, JG), postulated that an adapted a program could be useful in other complex health environments where the integration of different specialities is essential for delivering high‐quality comprehensive care. Therefore, this study describes the development of a new program of work, building on MDT‐FIT, undertaken beyond cancer services to develop a broader teamwork program, called Team Evaluation and Assessment Measure—Quality Improvement (TEAM‐QI). We aimed to determine the features and content that would be relevant for all (or most) types of healthcare teams and then test the proof of concept of the prototype program (TEAM‐QI) in maternity services as a first step of this adaptation. Maternity services were selected for the first area of adaption and testing as it provides a multidisciplinary, high‐stakes environment that allows us to evaluate the feasibility and functionality of the adapted program in a complex healthcare setting and due to the teamwork improvement need identified in recent enquiries (National Maternity Review 2016; Kirkup 2022; Ockenden 2022).

## 2 Materials and Methods

### 2.1 Design and Context

A multistage project was undertaken to develop TEAM‐QI. The project comprised: (i) development and adaptation (engagement with representatives, a rapid review, and content mapping) and (ii) a proof‐of‐concept study in maternity services. The design of the study was underpinned by the Consolidated Framework for Implementation Research (CFIR) which provides a comprehensive theoretical framework that helps to design, evaluate, and refine interventions. The CFIR, developed for health‐related interventions, allows systematic assessment and evaluation of factors that influence intervention implementation and effectiveness within complex systems (Damschroder et al. 2022). In this study, we used the CFIR to help guide intervention adaption and analyze the factors that might influence the successful implementation of TEAM‐QI in real‐world settings (Damschroder et al. 2022; Lam et al. 2021), as detailed below and in further detail provided in Table S2.

#### 2.1.1 Development and Adaption of Program

##### 2.1.1.1 Engagement With Representatives and Rapid Review

**FIGURE 1:**
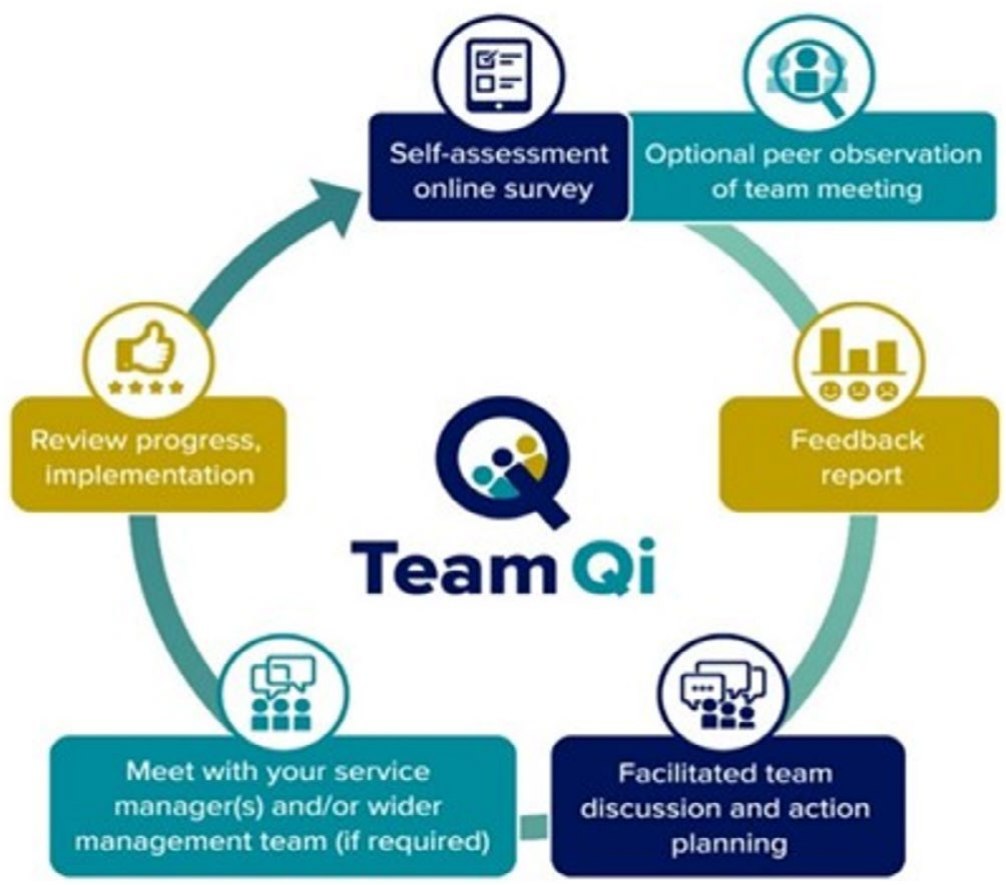
Proposed process for TEAM‐QI.

To identify the requirements for a program designed for all healthcare teams, online meetings with representatives (JH, CT) were held from February to March 2021. These representatives included individuals with prominence in developing and implementing national policy, frontline clinical staff at different levels of seniority across primary and secondary care, those with strategic or managerial roles in the NHS, representatives of independent regulators of health services, and subject matter experts including but not limited to maternity services. Representatives were approached via email through our professional networks/snowballing, using convenience sampling. The structure of the meetings consisted of an introduction to the MDT‐FIT program and the proposed adaptations. Open‐ended questions were used to enable the identification of salient issues informed by the CFIR framework (Damschroder et al. 2022). For additional detail, Table S3 provides the example topic discussion for meetings with representatives. Meetings with representatives covered issues in teamworking and culture, the perceived usefulness of the proposed program (prototype of TEAM‐QI: Figure 1, Tables 1 and S4), the engagement/leverage required for effective implementation and areas of readiness or resistance, areas of overlap with existing tools/programs, and potential context‐specific adaptions. In line with guidance (Collins et al. 2018), detailed logs were made during each meeting, mapping key issues and priorities of representatives. Implications for future design, practical issues, and wider impact were assessed to inform subsequent stages. Reflections were mapped (JH, CT) against the CFIR (Damschroder et al. 2022). Alongside this, a rapid literature review was undertaken to identify existing tools or programs (Harris et al. 2022), and feedback on the proposed program was provided by a local health services service user group.

**TABLE 1:** Explanation of the components of TEAM‐QI prototype intervention.

Process	Elaboration
Set date for the facilitated meeting	A facilitator is identified by the team with help from the organizational administrator. The team and facilitator agree a date/time/place for the facilitated meeting, usually at least 6–8 weeks in advance. Typically, these meetings are 60–90 min. With agreement, team and facilitator/observer details are added to the TEAM‐QI online platform and people are invited to register. See supplement 4 for further details of the facilitator and observer specification.
Self‐assessment online survey	All team members complete the validated Team Evaluation and Assessment Measure (Taylor et al. 2012) using a personalized email link to access the TEAM‐QI online platform. The survey takes about 8–10 min to complete. Automated reminders prompt non‐responders.
Optional peer observation of team meeting	For teams with a regular team meeting, a meeting can be observed by an independent observer using the validated Meeting Observational Tool (Harris et al. 2016). The date/time is agreed by all team members to ensure that it is a usual meeting (e.g., core members are not on leave), and the observer can choose to observe more than one meeting. Observations are entered online on the TEAM‐QI online platform. Automated reminders prompt observers to enter their assessment.
Feedback report	At least 1‐week before the facilitated meeting, the survey is closed, and the feedback report (synthesizing team member feedback from TEAM‐U and the observers report if completed) is automatically generated by the TEAM‐QI online platform. It is sent to all team members and the facilitator.
Facilitated team discussion and action planning	All team members attend the pre‐arranged facilitated meeting to discuss the feedback report; to identify strengths/what works well and to prioritize actions for improvement. Specific, Measurable, Achievable, Realistic and Time‐specific (SMART) actions are entered into the TEAM‐QI online platform and assigned to responsible individuals. SMART actions are categorized as to whether the team have the ability/resources to resolve them, or whether they require institution/organization support to implement. All actions (but not the feedback report) are shared with institution/organization management.
Meet with service manager/wider management team	If required, the team lead, facilitator and any other relevant team members meet with their management team to discuss their actions plans/develop implementation plan.
Review progress	The TEAM‐QI online platform prompts reminders for the implementation/updates on actions with the opportunity to review progress before launching new TEAM‐QI cycle.

##### 2.1.1.2 Content Mapping

Feedback from engagement with representatives and the rapid review were used to adapt the questionnaire, as well as inform the design of the online platform to ensure its suitability for all types of healthcare teams, beyond oncology. Updated or new items were then generated as statements with a 5‐point Likert response from strongly agree to strongly disagree (plus “don't know”). These were checked for clarity against the Questionnaire Assessment Scale (Willis and Lessler 1999) and reviewed by experts in questionnaire design (JH), organizational (CT) and health psychology (SB), midwifery (DB), and clinicians (JG, BL, MAA).

To understand the intervention attributes, evaluate theoretical underpinnings, and identify coverage and any gaps, the content of the resulting prototype questionnaire and the wider program (TEAM‐QI) were mapped to an evidence‐based framework of team effectiveness (the Integrated Team Effectiveness Model (ITEM)) (Lemieux‐Charles and McGuire 2006). The ITEM provides an overview of core, interconnecting dimensions of teamwork in healthcare settings. The ITEM was used as it enabled identification of the macro (health systems, social, and policy context), meso (organizational context including goals, structures, rewards), and micro (team) features that relate to team effectiveness and aligned with the approach used in our previous rapid review (Harris et al. 2022). The content was assessed independently (SB), then verified (JH and CT) with areas of uncertainty discussed, and agreed by consensus.

#### 2.1.2 Proof‐Of‐Concept Pilot of Questionnaire Component

##### 2.1.2.1 Procedure

The final stage of development was a proof‐of‐concept pilot of the questionnaire component of TEAM‐QI within a maternity setting. The original development of this questionnaire is outlined in another article, where psychometrics are detailed (Taylor et al. 2012). Four maternity teams were recruited from two NHS trusts in England. Information about the study was sent to the head of service at the Trusts who approached team leads about participation and subsequently met the authors (JH or JG) to discuss the study further before separately consulting their teams regarding their participation. Subsequently, all members of the four teams (*n* = 42) were invited by email to complete the questionnaire online (hosted on Qualtrics) including the new maternity‐specific items. To evaluate CFIR characteristics including intervention characteristics such as acceptability and appropriateness and readiness for implementation, respondents were asked about the perceived usefulness and content of the questionnaire, and to explain their answer. As a proof‐of‐concept pilot, sample sizes of 30–50 are considered sufficient for feasibility assessment and estimating standard deviations for future study design (Lancaster, Dodd, and Williamson 2004; Julious 2005).

##### 2.1.2.2 Data Analysis

To enable preliminary assessment of the attributes of the questionnaire (including CFIR intervention characteristics such as strength and quality and complexity), total scores were computed for the subscales, comprising of core (relevant to all types of healthcare teams), meeting (applicable to teams with regular team meetings) and maternity‐specific items. To assess feasibility, descriptive statistics including the frequency of endorsed items as an indicator of acquiescence bias (i.e., those choosing response options strongly agree and agree) and data quality (missing data, floor/ceiling effects and “don't know” responses) were undertaken as proof of concept of the scale. Internal consistency was assessed (Cronbach's alpha (α)) overall and for the effect of deleting individual items with values above 0.70. Average items covariance was also reported (Streiner, Norman, and Cairney 2015). For internal consistency, α values> 0.70 were considered good and accompanying average items covariance values 0.20–0.40 were indicative of moderate internal consistency (i.e., suggestive of values measuring a cohesive construct and no extremely high values which can indicate redundancy of items). Data were analyzed using Stata version 18 or higher. Open‐text responses were analyzed thematically (Neuendorf 2017).

##### 2.1.2.3 Ethics Statement

The study design and its materials were reviewed and received approval by an NHS ethics committee and the Health Research Authority (IRAS project ID:3000304/REC: 21/HRA/2461). All participants were provided with an information sheet and provided informed consent to take part in the study.

## 3 Results

### 3.1 Engagement With Representatives

#### 3.1.1 Acceptability of Program

Meetings with representatives took place with 14 professionals across multiple disciplines (midwifery/nursing, obstetrics, gynecological cancer, general practice) and organizations, and five members of healthcare service user groups. Representatives expressed a need for teamwork interventions that were team‐led, non‐punitive and based on facilitating cycles of continuous assessment and improvement. They also discussed key issues and priorities for team improvement and feedback on the proposed TEAM‐QI program (discussions with representatives referred to in the following section are summarized in Table 2).

**TABLE 2:** Overview of discussions with representatives.

Issues and priorities	Implications for TEAM‐QI design
1. Utility of TEAM‐QI Representatives and service users positive about usefulness and the aim of the tool. Representatives positive on underlying principles including self‐ and observer‐ assessments including free text entries, cycles of intervention, developmental not judgemental approach. Felt wording of measures was appropriate for use beyond cancer teams. Representatives advised that the language not be England‐centric, be aware that there may be some resistance (by individuals or organizations) which may impact implementation.	The most appropriate program cycle for TEAM‐QI that will suit most types of healthcare teams include: assessment and feedback, facilitated discussion and planning, followed by review. TEAM‐QI will focus on being team‐led but supported by the organization to reduce resistance. Wording on the platform, materials and measures to be more generic and refer to healthcare organizations beyond the NHS/Hospital trusts.
2. Teamwork, team membership and culture Teams are not only multidisciplinary (e.g., in maternity can be discipline specific), teams may have flatter hierarchies and team size can vary widely. Teams must also liaise with other healthcare teams and services which should be reflected in the survey. Representatives highlighted importance of assessing perceived ability to speak up in teams/dissent for patient safety. Covid‐19 has led to an increase in hybrid working which includes team meetings. Plus, it was noted that not all teams have regular meeting, nor would want their meeting observed.	The design of TEAM‐QI processes and online platform need to be flexible/applicable for different types of healthcare teams and membership (e.g., include unidisciplinary teams and multidisciplinary, include varying sizes of teams, multi‐site working). It should also be flexible to allow for teams to have the option to complete meeting items and to have their meetings observed.
3. Adaptability of TEAM‐QI Core items in the questionnaire component were considered appropriate for all types of teams, with some minor adaptions to wording required. Some additional items required regarding teamwork cultures, interprofessional services and services working, plus a set of maternity‐specific items were requested. Representatives felt the tool would be improved if teams could compare their effectiveness against aggregated scores of other similar teams in different organizations, and also compare their own effectiveness over time (through multiple cycles). Being able to track the implementation of actions on the platform was also valued, to increase organization support (e.g., if multiple teams have similar ‘issues’).	Adaptions based on feedback include: Add new items to the questionnaire (e.g., interprofessional working, engagement in learning and training) as well as maternity‐specific items Extend existing items in questionnaire (e.g., effectiveness of online/hybrid working, technology for hybrid/multi‐site working) Improve flexibility to different types of teams (not just MDT's, having the meeting items optional) Development data dashboards to allow for comparisons with aggregated team scores, to be able to track action implementation.
4. Strategic leadership implementation All representatives agreed senior managers need to support, engage and resource TEAM‐QI process for successful outcomes. To improve success, TEAM‐QI should link to national policy drivers. All supported actions being shared with organizational leads for strategic oversight (e.g., to be able to track progress on the data dashboards and help with bottlenecks). Interest in organizations adding ‘local’ items to the survey, but not too many to avoid participant burden.	Engagement of managers and clinical leaders will be key to the implementation of TEAM‐QI. To do this, we will develop materials and a short film. Dashboards will be designed to allow for action tracking (for individual team and organization‐wide participation of which they can compare). Organizations will be able to add up to three ‘local’ statements to the survey.
5. Awareness of similar other interventions Two teamwork interventions were identified by representatives, although most were not aware of other teamwork interventions.	Interventions included: SCORE tool (culture survey), and NOTSS (Non‐Technical Skills for Surgeons; technical skills rating tool). See Harris et al. (2022) for further details. There was minimal overlap/duplication with these tools and TEAM‐QI.

#### 3.1.2 Adaptions of the Program and Questionnaire

Suggested adaptations included ensuring that program terminology was generalizable to diverse professional populations and geographic regions. A key finding was that the TEAM‐QI process and online platform needed to be more flexible than MDT‐FIT to be relevant to different types of teams (e.g., in maternity, uni‐disciplinary as well as multidisciplinary teams might wish to use the program) and that supporting material needed to be less specific to England so it could be used in other regions of the UK and potentially internationally.

Furthermore, questionnaire items and the platform's functionality needed to be adaptable to hybrid working (meeting online and/or face to face), accommodate teams that work across multiple locations, services and levels of care, and teams that may not have regular team meetings. Adaptions ensured all core items of the questionnaire were generic and suitable for all types of teams (not cancer specific). Maternity representatives proposed that the questionnaire component be supplemented with additional items, specifically items that assessed: situational awareness, quality of handovers, working with other clinical teams, ensuring the service users voice is heard, and communication protocols in delivery suits/theater.

In relation to the specification of a future digital platform, representatives wanted to be able to compare their team performance to other “similar” teams (accepting that other team data would have to be aggregated to preserve anonymity) and wanted to track changes in team effectiveness over time (e.g., through multiple cycles of TEAM‐QI). Having the ability to track the implementation of actions was valued as an enabler for change in itself and also to facilitate getting the “buy‐in” and support from those with power within the organization. Representatives also stated that TEAM‐QI could help teams and organizations meet external pressures (e.g., national policy drivers) which may increase organizational support to implement change.

The rapid review (Harris et al. 2022) found that most teamwork interventions focused on micro features of teamwork (e.g. leadership, communication, decision‐making) without including relevant organizational and systems features and that many were task‐specific (e.g., improving safety in obstetric emergencies) rather than about teamworking across the whole care pathway. These findings and the meetings with representatives were used to refine the wording of statements in the questionnaire component (28 items), remove one item, and generate 12 new items universally relevant to healthcare teams (e.g., virtual teamworking, collegiality between professional groups, and organizational learning). Six additional items were created to reflect maternity‐specific aspects of teamwork. The prototype of this questionnaire (named TEAM‐U (Team Evaluation Assessment Measure—Universal)) included 39 “core” items (relevant to all healthcare teams), 19 “meeting” items (for use by teams that meet regularly), plus the 6 “maternity specific” items (Table S5 for exemplar items).

#### 3.1.3 Content Mapping

**FIGURE 2:**
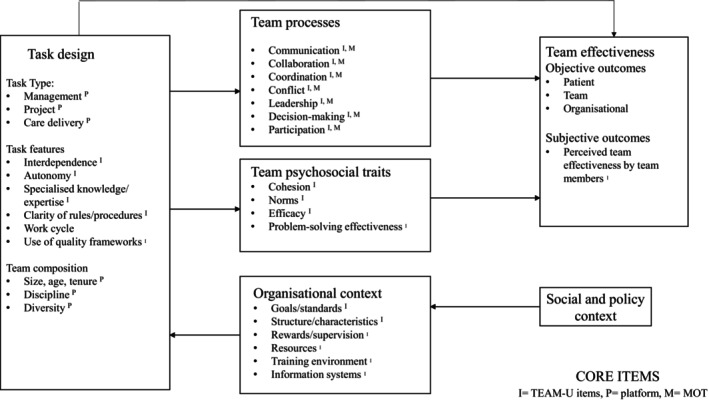
TEAM‐QIIntervention components mapped onto ITEM (Lemieux‐Charles and McGuire 2006).

The results of the TEAM‐QI program content mapping are shown in Figure 2, and an example of how content was coded is provided in Table S6. Overall, TEAM‐QI adhered to theoretical conceptualizations of team effectiveness (Lemieux‐Charles and McGuire 2006). Team processes (e.g., leadership communication, collaboration, coordination, etc.) were comprehensively covered by the questionnaire (TEAM‐U) and observer assessment components of the program. The questionnaire also enabled assessment of effectiveness in terms of psychosocial traits (such as cohesion, norms, efficacy, and problem‐solving) and the wider organizational context (e.g., by including items measuring perceptions about standards of care, resources, training, and equipment). Additionally, the questionnaire mapped onto subjective outcomes, measuring self‐reported assessments of team effectiveness in multiple items (e.g., team members assessments on the standard of care their team provides, and their enjoyment of working in their team). TEAM‐QI was not designed to measure impacts on teams from wider social and policy contexts, or to measure objective outcomes (though such data may be integrated into the facilitated discussion to inform actions for improvement).

### 3.2 Proof‐of‐Concept Pilot of TEAM‐U

#### 3.2.1 Characteristics and Participation

In the four teams (from two NHS Trusts), we recruited 26/42 team members (62%) to complete the online questionnaire. Response rates varied between teams (44%–86%). Respondents predominately included midwives (*n* = 21), with smaller representation from obstetricians (*n* = 2), pediatricians (*n* = 2), and an anesthetist (*n* = 1). Two of the teams consisted exclusively of midwives. One team did not have regular team meetings which precluded completion of the meeting‐related items and another composed of maternity management staff who also belonged to other clinical teams.

#### 3.2.2 Feasibility Assessment of TEAM‐U Properties

**FIGURE 3:**
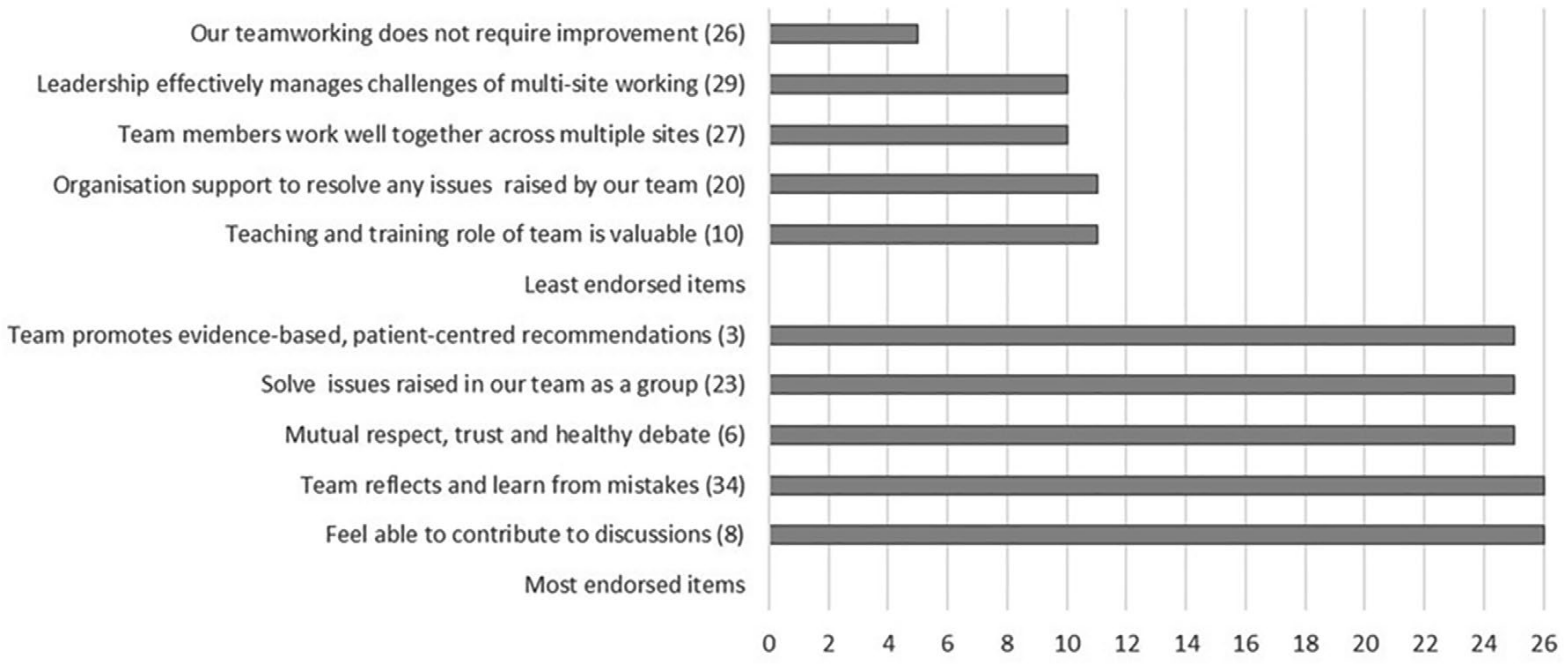
TEAM‐U core items most and least likely to be endorsed; shortened wording of item (position in questionnaire).

The TEAM‐U questionnaire covered core, meeting, and maternity modules. Details of item counts and score ranges are provided in Table 3 (CFIR intervention characteristics: adaptability). Missing responses were few, with four items having ≥ 5 missing responses, and “don't know” responses were infrequently chosen. Completion took a median of 20 min (CFIR intervention characteristics: complexity). The module scales had a modest positive skew (Table 3) and no floor/ceiling effects. High levels of internal consistency were observed (core items α = 0.92, meeting items α = 0.89, maternity items α = 0.79; see Tables S7.1–S7.3), with moderate inter‐item relationships (CFIR intervention characteristics: strengths and qualities). Most team members agreed on key positive aspects of team functioning such as feeling able to contribute to discussions, promoting evidence‐based patient care, and learning from mistakes (Figure 3). However, areas of challenge included effectively managing multisite working, organizational support in resolving issues, and the educational development role of the team (Figure 3).

**TABLE 3:** Preliminary assessment of TEAM‐U data quality.

Organization and team	Median total score (IQR)	Observed range of total scores	Mean total score (SD)	Median (IQR) number of endorsed items^1,2^	Median (range) items with “don't know” responses
TEAM‐U core module					
Organization 1	141.0 (130, 154)	119–188	144.3 (20.90)	28 (23, 32.5)	0.5 (0–7)
Team 1 (*n* = 7)	143.0 (139, 149)	128–187	147.3 (18.66)	28 (22, 30)	0 (0–7)
Team 2 (*n* = 9)	132.0 (126, 158)	119–188	141.9 (23.32)	30 (23, 33)	1 (0–7)
Organization 2	152.0 (141, 157)	112–167	147.0 (17.47)	29.5 (25, 32)	0 (0, 10)
Team 3 (*n* = 6)	156.5 (151, 162)	149–167	157.0 (6.72)	31 (29, 32)	0 (0–4)
Team 4 (*n* = 4)	131.5 (117, 147)	112–153	132.0 (18.46)	24.5 (19, 31.5)	4 (0–10)
All	143.6 (131, 157)	122–188	145.3 (19.34)	28.5 (23–32)	0 (0–10)
TEAM‐U meeting module					
Organization 1	67.0 (63, 77)	55–87	69.8 (9.67)	14 (10.5, 16)	0 (0–1)
Team 1 (*n* = 7)	64.0 (58,78)	55–85	68.4 (11.27)	11 (8, 17)	0 (0–2)
Team 2 (*n* = 9)	69.0 (64, 76)	60–87	70.8 (8.80)	14 (12, 16)	0 (0–1)
Organization 2	—	—	—	—	—
Team 3 (*n* = 6)	—	—	—	—	—
Team 4 (*n* = 4)	61.5 (56, 71)	54–75	63.0 (9.49)	10 (8, 14.5)	0 (0–1)
All	63.5 (54,75)	54–86	52.6 (30.60)	11.5 (6,15)	0 (0–2)
TEAM‐U maternity module					
Organization 1	16.5 (14, 24)	12–30	18.9 (6.13)	4.5 (3, 6)	0 (0, 2.5)
Team 1 (*n* = 7)	17 (12, 23)	12–29	18.1 (6.49)	3 (3, 4)	0 (0–3)
Team 2 (*n* = 9)	16 (14, 25)	13–30	19.4 (6.17)	6 (5, 8)	0 (0–7)
Organization 2	24 (21, 25)	19–29	23.5 (3.50)	4 (1,6)	0 (0–1)
Team 3 (*n* = 6)	22 (21, 25)	19–28	22.8 (3.25)	4 (4, 4)	0 (0–1)
Team 4 (*n* = 4)	25 (22, 27)	19–29	24.5 (4.12)	6 (4.5, 6)	0 (0)
All	21 (16, 25)	12–30	20.7 (5.68)	4 (3, 6)	0 (0–7)

*Note:* 1 Median and IQR unless otherwise specified; 2number of items where response was (strongly)agree.

Abbreviations: IQR, interquartile range; SD, standard deviation.

### 3.3 Acceptability of TEAM‐U

Most respondents (76.9%) considered TEAM‐U would be useful for identifying priorities for improvement, with detailed feedback (16/26 team members) highlighting the importance of inclusivity, comprehensiveness and its potential to support a diagnostic and interventional approach for team improvement (Table S8) (CFIR intervention characteristics: strengths and qualities). A minority (4/16 team members) questioned the relevance of certain items to their particular team (e.g., those more relevant to inpatient or labor wards, where they worked in community settings) and commented that the utility depends on subsequent application of findings and how they are used by the team (3/16 team members; CFIR intervention characteristics: readiness for implementation).

## 4 Discussion

This study outlined the development of TEAM‐QI, a program designed to enhance team performance in healthcare settings. The design and adaption process involved multiple stages to ensure that TEAM‐QI was evidence‐based, incorporated input from representatives, addressed gaps in teamwork interventions, and covered key elements important for team effectiveness (Harris et al. 2022; National Cancer Action Team 2010; Lemieux‐Charles and McGuire 2006). Engagement with representatives was critical in shaping the development of TEAM‐QI, ensuring it could address the unique challenges of different healthcare environments and team structures. The feedback emphasized the need for flexibility in the program's implementation, particularly regarding virtual and hybrid teamworking, which has become increasingly relevant post‐pandemic (Groothuizen et al. 2023). The inclusion of maternity‐specific items and the adaptation to accommodate different professional groups also reflect the need for tailored approaches in healthcare teamwork interventions.

The resulting TEAM‐QI prototype, adapted from MDT‐FIT (Taylor et al. 2021), comprises a questionnaire assessment, observational component and feedback report (generated via an online platform). This feedback report serves as the basis of a facilitated discussion, during which team members agree on specific action plans. Initial testing of the questionnaire component (TEAM‐U) with maternity teams demonstrated its acceptability and feasibility. The high internal consistency of the questionnaire and positive feedback from most participants provide preliminary evidence that TEAM‐U is both reliable and comprehensive in capturing key aspects of team dynamics and performance. Further research is needed to validate its use at scale in different contexts and to refine its components for broader application in the healthcare system.

### 4.1 Interpretation Within the Context of the Wider Literature

Literature has highlighted that existing teamworking tools often solely focus on micro‐level tasks undertaken by teams (e.g., improving skills in emergency situations), ignoring the impact of wider organizational and systems contextual factors on team effectiveness, such as communication and coordination of care (Harris et al. 2022). Previous research has also highlighted the need for teamwork interventions that support evaluation and improvement both within and between teams (West and Lyubovnikova 2013). TEAM‐QI addresses these gaps by incorporating organizational and systems features, as well as micro “team” based features of teamwork, while also being designed to focus on the whole care pathway. This was demonstrated in the content mapping to the ITEM (Lemieux‐Charles and McGuire 2006), where TEAM‐QI mapped well onto the factors known to influence team effectiveness. The early stages of development of TEAM‐QI, underpinned by existing research and theoretical frameworks of effective teamworking (National Cancer Action Team 2010; Lemieux‐Charles and McGuire 2006) and key priorities highlighted by representatives, are in line with the development of other healthcare teamwork programs/tools (e.g., TeamStepps and Moreob) (King et al. 2008; Milne and Lalonde 2007).

### 4.2 Strengths and Limitations

The design of TEAM‐QI was informed by an evidence‐based implementation framework (Damschroder et al. 2022), engagement with representatives, and assessed against a model of team effectiveness (Lemieux‐Charles and McGuire 2006). Each stage of development ensured TEAM‐QI was underpinned by evidence regarding the key components of teamworking known to impact outcomes for patients, staff, and organizations (National Cancer Action Team 2010). The TEAM‐QI program itself serves to flatten hierarchies and give all team members a “voice” to speak up about their team strengths and challenges. Further strengths include that the team “owns” their data, thereby supporting a bottom‐up developmental rather than top‐down judgemental process, strengthening the likelihood of adoption and implementation effectiveness. It was beyond the scope of this preliminary study to investigate specific strategies (Powell et al. 2015) that lead to quality improvement within the TEAM‐QI program. However, it represents an important area for future research to evaluate its utility and potential benefits in greater depth.

Although TEAM‐QI has been designed for use in all healthcare settings, testing in this study was limited to a small number of maternity teams and has not evaluated its utility beyond this context. However, by adopting a phased approach we have sought to develop iterative improvements in the program to build a robust foundation for future testing in more diverse healthcare settings beyond maternity and oncology. Owing to this small sample size, analysis of the factor structure of the questionnaire was not possible and requires further validation. It should also be acknowledged that we did not collect demographic information on representatives and participants, only their professional background. As this was a proof‐of‐concept study, we focused on the feasibility and acceptability of the program. Future research should next look to select specific outcome variables associated with team effectiveness to assess the program. For the content mapping, ITEM (Lemieux‐Charles and McGuire 2006) was used to evaluate the content of TEAM‐QI which indicated good coverage of key components of team effectiveness. The ITEM was selected compared to the alternative, more generic frameworks such as a complex adaptive systems approach (Salas, Sims, and Burke 2005), the relational coordination framework (Gittel 2006), or the Big Five (Plsek and Greenhalgh 2001), as it was specifically developed based on literature about healthcare team effectiveness, has applicability for a range of healthcare settings, and was used previously in our rapid review (Harris et al. 2022). However, we acknowledge that other teamwork frameworks could have been used. While this provided a pragmatic and structured starting point for evaluating the content of TEAM‐QI, future research should incorporate elements from other models to understand and address potential gaps in TEAM‐QI.

When piloting the questionnaire (TEAM‐U), almost two‐thirds of team members responded to the online survey. This might be due to the questionnaire component being piloted alone, without the other features of TEAM‐QI, impacting on motivation to participate. At a maximum of 66 items (including open‐text questions), the length of the questionnaire may be a limitation at this time. Developing and testing a reduced scale may be beneficial for the program. It should be noted that the timing of piloting TEAM‐U also coincided with the tail end of the COVID‐19 pandemic in the UK (July to October 2021) when healthcare systems faced many challenges including staff ill‐health. Despite these limitations, team members commented that it was worthwhile and would benefit their teams.

### 4.3 Implications for Policy, Practice, and Research

The need to ensure healthcare teams can work effectively is a stated priority by both UK and international governments (Australian Commission for Safety and Quality in Health Care 2020; National Maternity Review 2016; UK Government 2023). If further supported through evaluation, TEAM‐QI may be part of a solution to meet this expressed need, particularly through addressing gaps highlighted in existing tools (Harris et al. 2022). In relation to practice, TEAM‐QI is designed to take as minimal time as possible, taking less than two hours for team members to participate to the point of agreeing actions (15‐ to 20‐min survey, 30‐min reading the feedback report, one‐hour facilitated discussion) (Taylor et al. 2021), so has the potential to confer benefits with minimal resource.

Where respondents questioned the specificity of items included, future iterations could include either a “not applicable” option or greater personalization to the type of maternity team (e.g., community, inpatient/acute). Future research will also include the development of specific/specialized modules for teams in other clinical areas of healthcare and settings. As this was a proof‐of‐concept study, the next steps will explore the above, while piloting the full TEAM‐QI program with larger samples of healthcare teams and include outcomes measures of team effectiveness. Assessment of the psychometric properties of the updated TEAM‐U questionnaire will also be conducted in addition to developing a shortened version.

## 5 Conclusion

This study described the development of TEAM‐QI, a program designed to improve team effectiveness through a structured evidence‐based approach. The development process incorporated engagement with representatives, emphasizing flexibility and tailoring for diverse settings and professional groups. Initial testing of TEAM‐U, the questionnaire component, demonstrated preliminary proof of concept (feasibility and acceptability), with early evidence suggesting its potential to capture important aspects of team performance. Future research should explore the scalability of TEAM‐QI, its adaptability to different clinical areas and healthcare settings, and its longer‐term impact on team performance and patient outcomes.

## Author Contributions

**Sarah Beck:** investigation, formal analysis, data curation, writing – original draft, project administration, methodology. **Jenny Harris:** conceptualization, methodology, investigation, formal analysis, supervision, funding acquisition, writing – original draft, writing – review and editing. **James Green:** conceptualization, methodology, writing – review and editing, funding acquisition. **Benjamin W. Lamb:** writing – review and editing, methodology. **Mehrnoosh Aref‐Adib:** writing – review and editing, methodology. **Debra Bick:** writing – review and editing, methodology. **Cath Taylor:** conceptualization, methodology, formal analysis, writing – original draft, writing – review and editing, supervision, funding acquisition, investigation.

## Ethics Statement

The study design and its materials were reviewed and received approval by an NHS ethics committee and the Health Research Authority (IRAS project ID:3000304/REC: 21/HRA/2461).

## Conflicts of Interest

Cath Taylor, Jenny Harris, and James Green have previously received funding from NHS organizations within England and Scotland for supporting cancer teamwork improvement; James Green is the Director of Green Cross Medical Ltd. that developed MDT‐FIT for use by National Health Service Cancer Teams in the UK. The other authors declare no conflicts of interest.

## Supporting information


Data S1

## Data Availability

The data that support the findings of this study are available from the corresponding author upon reasonable request.

## References

[nhs70049-bib-0001] Anderson, N. R. , and M. A. West . 1998. “Measuring Climate for Work Group Innovation: Development and Validation of the Team Climate Inventory.” Journal of Organizational Behaviour: The International Journal of Industrial, Occupational and Organizational Psychology and Behaviour 19: 235–258.

[nhs70049-bib-0002] Australian Commission for Safety and Quality in Health Care . 2020. “ Communicating for Safety: Improving Clinical Communication, Collaboration and Teamwork in Australian Health Services. Scoping Paper .” Accessed September 27,2023. https://www.safetyandquality.gov.au/sites/default/files/2020‐12/final_scoping_paper_‐_improving_communication_collaboration_and_teamwork_in_australian_health_services_‐_june_2020.pdf.

[nhs70049-bib-0003] Collins, M. , R. Long , A. Page , J. Popay , and F. Lobban . 2018. “Using the Public Involvement Impact Assessment Framework to Assess the Impact of Public Involvement in a Mental Health Research Context: A Reflective Case Study.” Health Expectations 21: 950–963. 29696740 10.1111/hex.12688 PMC6250886

[nhs70049-bib-0004] Damschroder, L. J. , C. M. Reardon , M. A. O. Widerquist , and J. Lowery . 2022. “The Updated Consolidated Framework for Implementation Research Based on User Feedback.” Implementation Science 17: 1–16. 36309746 10.1186/s13012-022-01245-0 PMC9617234

[nhs70049-bib-0037] Department of Health and Social Care . 2023. Press Release: Government Acts to Boost the Quality of Care for Mothers and Babies. UK Government Accessed September 6, 2023. https://www.gov.uk/government/news/government‐acts‐to‐boost‐the‐quality‐of‐care‐for‐mothers‐and‐babies.

[nhs70049-bib-0005] Francis, R. 2013. “ Report of the Mid Staffordshire NHS Foundation Trust Public Inquiry – Executive Summary .” Accessed September 6, 2023. https://assets.publishing.service.gov.uk/government/uploads/system/uploads/attachment_data/file/279124/0947.pdf.

[nhs70049-bib-0006] Gittel, J. H. 2006. “Relational Coordination: Coordinating Work Through Relationships of Shared Goals, Shared Knowledge and Mutual Respect.” In Relational Perspectives in Organizational Studies, edited by O. Kyriakidou and M. Ozbilgin , 74–94. Cheltenham, UK: Edward Elgar.

[nhs70049-bib-0007] Greenhalgh, T. , and C. Papoutsi . 2018. “Studying Complexity in Health Services Research: Desperately Seeking an Overdue Paradigm Shift.” BMC Medicine 16: 95. 10.1186/s12916-018-1089-4. 29921272 PMC6009054

[nhs70049-bib-0008] Groothuizen, J. E. , E. Aroyewun , M. Zasada , J. Harris , M. Hewish , and C. Taylor . 2023. “Virtually the Same? Examining the Impact of the COVID‐19 Related Shift to Virtual Lung Cancer Multidisciplinary Team Meetings in the UK National Health Service: A Mixed Methods Study.” BMJ Open 13: e065494. 10.1136/bmjopen-2022-065494 PMC10276952 37328174

[nhs70049-bib-0009] Harris, J. , S. Beck , N. Ayers , et al. 2022. “Improving Teamwork in Maternity Services: A Rapid Review of Interventions.” Midwifery 108: 103285. 35228116 10.1016/j.midw.2022.103285

[nhs70049-bib-0010] Harris, J. , C. Taylor , N. Sevdalis , R. Jalil , and J. S. A. Green . 2016. “Development and Testing of the Cancer Multidisciplinary Team Meeting Observational Tool (MDT‐MOT).” International Journal for Quality in Health Care 28: 332–338. 27084499 10.1093/intqhc/mzw030 PMC5892160

[nhs70049-bib-0011] Julious, S. A. 2005. “Sample Size of 12 per Group Rule of Thumb for a Pilot Study.” Pharmaceutical Statistics 4: 287–291.

[nhs70049-bib-0012] King, H. B. , J. Battles , D. P. Baker , et al. 2008. “TeamSTEPPS™: Team Strategies and Tools to Enhance Performance and Patient Safety.” In Advances in Patient Safety: New Directions and Alternative Approaches, Performance and tools, edited by K. Henriksen , J. B. Battles , M.A. Keyes and M.L. Grady , vol. 3. Rockville, MD (US).

[nhs70049-bib-0013] Kirkup, B. 2022. “ Reading the Signals: Maternity and Neonatal Services in East Kent – The Report of the Independent Investigation .” Accessed September 6, 2023. https://assets.publishing.service.gov.uk/government/uploads/system/uploads/attachment_data/file/1111992/reading‐the‐signals‐maternity‐and‐neonatal‐services‐in‐east‐kent_the‐report‐of‐the‐independent‐investigation_print‐ready.pdf.

[nhs70049-bib-0014] Lam, H. , M. Quinn , T. Cipriano‐Steffens , et al. 2021. “Identifying Actionable Strategies: Using Consolidated Framework for Implementation Research (CFIR)‐informed Interviews to Evaluate the Implementation of a Multilevel Intervention to Improve Colorectal Cancer Screening.” Implementation Science Communication 57: 2. 10.1186/s43058-021-00150-9 PMC8167995 34059156

[nhs70049-bib-0015] Lamb, B. W. , K. F. Brown , K. Nagpal , C. Vincent , J. S. A. Green , and N. Sevdalis . 2011. “Quality of Care Management Decisions by Multidisciplinary Cancer Teams: A Systematic Review.” Annals of Surgical Oncology 18: 2116–2125. 21442345 10.1245/s10434-011-1675-6

[nhs70049-bib-0016] Lancaster, G. A. , S. Dodd , and P. R. Williamson . 2004. “Design and Analysis of Pilot Studies: Recommendations for Good Practice.” Journal of Evaluation in Clinical Practice 10: 307–312. 15189396 10.1111/j..2002.384.doc.x

[nhs70049-bib-0017] Lemieux‐Charles, L. , and W. L. McGuire . 2006. “What Do We Know About Health Care Team Effectiveness? A Review of the Literature.” Medical Care Research and Review 63: 263–300. 16651394 10.1177/1077558706287003

[nhs70049-bib-0018] LePine, J. A. , R. F. Piccolo , C. L. Jackson , et al. 2008. “A Meta‐Analysis of Teamwork Processes: Tests of a Multidimensional Model and Relationships With Team Effectiveness Criteria.” Personnel Psychology 61: 273–307.

[nhs70049-bib-0019] Manser, T. 2009. “Teamwork and Patient Safety in Dynamic Domains of Healthcare: A Review of the Literature.” Acta Anaesthesiologica Scandinavica 53: 143–151. 19032571 10.1111/j.1399-6576.2008.01717.x

[nhs70049-bib-0020] Mazurenko, O. , J. Richter , A. Swanson‐Kazley , and E. Ford . 2016. “Examination of the Relationship Between Management and Clinician Agreement on Communication Openness, Teamwork, and Patient Satisfaction in the US Hospitals.” Journal of Hospital Administration 5: 20–27.

[nhs70049-bib-0021] Milne, J. K. , and A. B. Lalonde . 2007. “Patient Safety in Women's Health‐Care: Professional Colleges Can Make a Difference. The Society of Obstetricians and Gynaecologists of Canada MOREOB Program.” Best Practice & Research Clinical Obstetrics & Gynaecology 21: 565–579. 17376746 10.1016/j.bpobgyn.2007.01.013

[nhs70049-bib-0022] National Cancer Action Team . 2010. “ The Characteristics of an Effective Multidisciplinary Team .” Accessed October 3, 2023. http://www.ncin.org.uk/cancer_type_and_topic_specific_work/multidisciplinary_teams/mdt_development.

[nhs70049-bib-0023] National Maternity Review . 2016. “Better births: *Improving Outcomes of Maternity Services in England. A Five Year Forward View of Maternity Care* .” Accessed September 27, 2023. https://www.england.nhs.uk/wp‐content/uploads/2016/02/national‐maternity‐review‐report.pdf.

[nhs70049-bib-0024] Neuendorf, K. A. 2017. The Content Analysis Guidebook. Los Angeles: Sage.

[nhs70049-bib-0025] NHS England . 2023. “ Three Year Delivery Plan for Maternity and Neonatal Services .” Accessed September 6, 2023. https://www.england.nhs.uk/long‐read/three‐year‐delivery‐plan‐for‐maternity‐and‐neonatal‐services/#theme‐4‐standards‐and‐structures‐that‐underpin‐safer‐more‐personalised‐and‐more‐equitable‐care.

[nhs70049-bib-0026] NHS Staff Survey . 2022. “ NHS Staff Survey National Results Briefing .” Accessed September 6, 2023. https://www.nhsstaffsurveys.com/results/national‐results/.

[nhs70049-bib-0027] Ockenden, D. 2022. “ Findings, Conclusions and Essential Actions From the Independent Review of Maternity Services at the Shrewsbury and Telford Hospital NHS Trust .” Accessed September 6, 2023. https://assets.publishing.service.gov.uk/government/uploads/system/uploads/attachment_data/file/1064302/Final‐Ockenden‐Report‐web‐accessible.pdf.

[nhs70049-bib-0028] Plsek, P. E. , and T. Greenhalgh . 2001. “Complexity Science: The Challenge of Complexity in Health Care.” BMJ 323: 625–628. 11557716 10.1136/bmj.323.7313.625 PMC1121189

[nhs70049-bib-0029] Powell, B. J. , T. J. Waltz , M. J. Chinman , et al. 2015. “A Refined Compilation of Implementation Strategies: Results From the Expert Recommendations for Implementing Change (ERIC) Project.” Implementation Science 10: 21. 25889199 10.1186/s13012-015-0209-1 PMC4328074

[nhs70049-bib-0030] Ramanujam, R. , and D. M. Rousseau . 2006. “The Challenges Are Organizational Not Just Clinical.” Journal of Organizational Behaviour: The International Journal of Industrial, Occupational and Organizational Psychology and Behaviour 27: 811–827.

[nhs70049-bib-0031] Salas, E. , D. E. Sims , and C. S. Burke . 2005. “Is There a “Big Five” in Teamwork?” Small Group Research 36: 555–599.

[nhs70049-bib-0032] Schmutz, J. , and T. Manser . 2013. “Do Team Processes Really Have an Effect on Clinical Performance? A Systematic Literature Review.” British Journal of Anaesthesia 110: 529–544. 23454826 10.1093/bja/aes513

[nhs70049-bib-0033] Schmutz, J. B. , L. L. Meier , and T. Manser . 2019. “How Effective Is Teamwork Really? The Relationship Between Teamwork and Performance in Healthcare Teams: A Systematic Review and Meta‐Analysis.” BMJ Open 9: e028280. 10.1136/bmjopen-2018-028280 PMC6747874 31515415

[nhs70049-bib-0034] Streiner, D. L. , G. R. Norman , and J. Cairney . 2015. Health Measurement Scales: A Practical Guide to Their Development and Use. Oxford: Oxford University Press.

[nhs70049-bib-0035] Taylor, C. , K. Brown , B. Lamb , J. Harris , N. Sevdalis , and J. S. A. Green . 2012. “Developing and Testing TEAM (Team Evaluation and Assessment Measure), a Self‐Assessment Tool to Improve Cancer Multidisciplinary Teamwork.” Annals of Surgical Oncology 19: 4019–4027. 22820934 10.1245/s10434-012-2493-1

[nhs70049-bib-0036] Taylor, C. , J. Harris , K. Stenner , N. Sevdalis , and J. S. A. Green . 2021. “A Multi‐Method Evaluation of the Implementation of a Cancer Teamwork Assessment and Feedback Improvement Programme (MDT‐FIT) Across a Large Integrated Cancer System.” Cancer Medicine 10: 1240–1252. 33480191 10.1002/cam4.3719 PMC7926008

[nhs70049-bib-0038] West, M. A. , and J. Lyubovnikova . 2013. “Illusions of Team Working in Health Care.” Journal of Health Organization and Management 27: 134–142. 23734481 10.1108/14777261311311843

[nhs70049-bib-0039] Willis, G. B. , and J. T. Lessler . 1999. Question Appraisal System QAS‐99: Guidelines for Designing and Evaluating Questionnaires. Rockville, MD: Research Triangle Institute.

[nhs70049-bib-0040] Zajac, S. , A. Woods , S. Tannenbaum , E. Salas , and C. L. Holladay . 2021. “Overcoming Challenges to Teamwork in Healthcare: A Team Effectiveness Framework and Evidence‐Based Guidance.” Frontiers in Communication 6: 606445.

