# Supplementary material for: Development of the Team Evaluation and Assessment Measure Quality Improvement (TEAM‐QI) and Proof‐Of‐Concept Testing in Maternity Teams
Source: Nurs Health Sci. 2025 Feb 3;27(1):e70049. doi: 10.1111/nhs.70049 (PMC11790520; doi:10.1111/nhs.70049)
Supplement: Supplementary file 1 — Data S1 [file NHS-27-e70049-s001.docx]

Supplementary materials:

**Supplementary Tables:**

**Supplementary Table 1: Summary of Buljac et al (2020) classification of intervention types^*^**

| **Intervention type** | **Description (and subcategories)** |
| --- | --- |
| Training | Training to improve teamwork. Subcategories include principle-based training, simulation-based training, and general team training. |
| Tools | Instruments implemented relatively independently in order to structure, facilitate or trigger teamwork |
| Organisational (re)design | Interventions to change organisational structure for example in the payment system, changing of roles or the formation of teams. |
| Programme | Consists of a ‘bundle’ that combines learning and educational sessions, tools and/or structural changes. It frequently takes the organisational context into account. |

^*^Extracted and adapted from: Buljac-Samardzic, M., Doekhie, K. D., & van Wijngaarden, J. D. H. (2020). Interventions to improve team effectiveness within health care: A systematic review of the past decade. *BMC Human Resources for Health, 18*(2).

**Supplementary Table 2: How Consolidated framework for implementation research (CFIR) informed the intervention and study design**

| **CFIR use in adapting the intervention** | **Examples of CFIR application in study design** |
| --- | --- |
| **Comprehensive Assessment**: CFIR provides a structured way to assess all relevant factors that could affect implementation success. | Informed consultation with representatives content by allowing for a thorough understanding of potential barriers and facilitators.  This was primarily focused on intervention characteristics (attributes of the programme, such as complexity, adaptability and preliminary testing of the TEAM-U questionnaire); outer setting (influences like patient needs, resources, and policies that can impact implementation); inner setting (Internal factors within the organisation, including culture, climate, and readiness for implementation) |
| **Guiding Intervention Design**: By identifying the specific contexts and conditions that are critical to implementation. | Information from consultation with representatives and the content of the prototype intervention was mapped to CFIR which helps tailor interventions to fit the setting and the needs of the target population. Additionally, the intervention was mapped to ITEM to evaluate theoretical underpinnings. |
| **Facilitating Adaptation**: The framework supports the adaptation of interventions to different settings or populations by highlighting which aspects of the intervention or context need modification. | The TEAM-U questionnaire was adapted based on the literature review and mapping of reflections from engagement with representatives. Six additional items were created to reflect maternity specific aspects of teamwork and meeting items were made optional as not all teams will have a regular team meeting. |
| **Improving Implementation Success**: By addressing the key factors identified through CFIR, intervention designers can enhance the likelihood of successful implementation, sustainability, and ultimately, the effectiveness of the intervention in achieving its goals. | Information from consultation with representatives and the content of the prototype intervention was mapped to CFIR which helps tailor interventions to fit the setting and the needs of the target population. |

**Supplementary Table 3: Example of topic discussion prompts for meetings with representatives**

| **Introductions** and start with an overview of TEAM-QI prototype and purpose of the work  Confirm okay to record/confidential (anonymous), explain not ‘research’ but to write summary - if not okay take detailed notes  **PRIMARILY CLINICAL/CLINICAL LEADERS:**  **Usefulness:** could this be useful [in your area? Why or why not? If useful, where could it fit?  **Need:** What do you see as the main issues teamwork that affect patient care/staff wellbeing (positive or negative)?  **Appropriateness:** Find out what definition their definition of team is and what they think we should include  Can you tell me about the different teams you are part of and work with in [specific area] that could use TEAM QI?  In these different teams do you meet formally in any format (e.g. would the observational component be something that could be used)?  What is the membership/key players in each team?  Probe: are they formerly named teams (reg staff meetings)/ or more informal teams?  If unclear: are formal MDT meetings (clinical decision-making) held regularly?  If identify key couple team or teams: We are interested in understanding how different teams work together: who are the teams your team work with in order to deliver patient care?  **Culture/readiness for** change: do you think people would be open to something like TEAM QI? Probe: likely resistance? To what extent are new ideas embraced and used to make improvements in your organisation?  **Adaptability of TEAM QI (MDT-FIT): Are there any changes or alterations do you think we will need to make to MDT-FIT so it will work effectively in your setting?** Discuss any **changes re: process (Trust management overseeing; independent facilitator and observer) and content (ask if we can send them TEAM for review; check re: appropriateness of observational component).**   - - Why or why not? (probe re appropriateness of observation if not covered)   - Are there components that should not be altered?   **PRIMARILY POLICY/STRATEGIC/ INCLUDING NON-CLINICAL LEADERS:**  **Usefulness:** could this be useful [in your area? Why or why not? If useful, where could it fit?  **Need:** What do you see as the main issues teamwork that affect patient care/staff wellbeing (positive or negative)? (Probe re fit/appropriateness): what are the strategic priorities/policy drivers relating to teamwork? And how are these currently being addressed/tackled?  **Culture/readiness for** change: do you think people would be open to something like TEAM QI? Probe: likely resistance? To what extent are new ideas embraced and used to make improvements in your organisation?  **DICUSSION POINTS FOR ALL**  **Who would need to be involved to make TEAM QI work in [your setting/clinical area]? (leadership/engagement/staff)**  **Leverage/meeting needs:** To what extent would implementing TEAM QI provide an advantage [for your organisation] compared [to other organisations] in your area? Probe: local or national performance measures, policies, regulations, or guidelines that might influenced the decision  **Are you aware of any similar tools/interventions being used in your area?** Probe: can you describe it? How does it compare? (try to determine relative advantage/needs/resources) Probe if have any strategic or planned annual team review processes, currently.  **Key influential individuals to get on board (opinion leaders, champions, representatives, grassroots):** Who are the key individuals to get on board with the intervention? Other than the formal implementation leader, are there people in [your organisation] who are likely to champion (go above and beyond what might be expected) the intervention? How do we ‘sell it’ to team leaders and members?  Share **content of TEAM questionnaire**- to see if anything they think is important is missing |
| --- |

**Supplementary Table 4: Explanation of key roles in Team-QI external to the team lead/members**

| **Roles** | **Elaboration** |
| --- | --- |
| **Rationale for external roles** | TEAM-QI encourages organisational learning by utilising institution/organisation staff (clinicians and managers) to act as independent observers and facilitators, and to help teams run/organise the process (see organisational administrator). |
| Facilitator | The facilitator is agreed by the team leadership and should be a: i) a respected **Senior organisation/hospital** **colleague** (although they can be external to the organisation/hospital if preferred),  ii) Be a **senior clinician or manager,** with experience of working in or with clinical care teams/MDTs (e.g., they may work in a different area or service);  iii) **external to the team** and its immediate day-to-day functioning (i.e., not responsible for their management) to be independent – and to be seen as independent by the team;  iv) **confident and experienced at facilitating meetings** involving a diverse mix of health professionals; utilise the resources provided to help support the team (team feedback report, facilitators guide)  The facilitator should **NOT be:**  **i)** a member of the team or work with or alongside the team on a day-to-day basis;  ii) significantly more junior than the most senior member of the team;  iii) managed or accountable to a member of the team or manage anyone within the team.  **Materials**: provided with guides to facilitation. |
| Observer | The observer is agreed by the team leadership and should be: i) a respected Senior organisation/hospital colleague (although they can be external to the organisation/hospital if preferred);  ii) a senior clinician or manager, with experience of working in or with clinical care team/MDTs (e.g., they may work in a different area or service);  iii) external to the team and its immediate day-to-day functioning (i.e., not responsible for their management) in order to be independent – and to be seen as independent by the team;  iv) be confident in their ability to assess the performance of team in meetings using a structured tool provided; familiar with the diverse mix of health professionals, typical characteristics of such meetings (e.g., terminology used, types of tests discussed, and format of meetings).  The observer should NOT be: i) a member of the team or work with or alongside the team on a day-to-day basis;  ii) significantly more junior than the most senior member of the team,  iii) managed or accountable to a member of the team or manage anyone within the team.  **Materials:** provided with the meeting observational tool and rating guide |
| Organisational administrator | Help team run the process (e.g. identify possible facilitators/observers, room booking/sending emails and setting the team up on the Team-QI online platform). |

**Supplementary Table 5: Example of TEAM-U items**

| **Section of questionnaire (TEAM-U)** | **Sample TEAM-U items** |
| --- | --- |
| Core items (Team) | I would like my team to look after a family member or friend if they needed their service.  Leadership is effective in our team.  I feel part of a first-class team.  The organisation provides adequate technology for teamwork (e.g., ability to share information from multiple sources) |
| Meeting items | If the patient is not present, someone who has met the patient attends to discuss their case.  I am able to attend our team meeting regularly, and this is reflected in my job plan.  Clinical information is presented to a high standard at our team meetings.  The minimum amount of information required for each case/issue is agreed by the team (in its guidelines). |
| Maternity module | Our team ensures that women and their families are listened to and their voices heard.  In the delivery suite/theatre our team makes good use of structured communication protocols (e.g. checklists, closed-loop communication) |

**Supplementary Table 6: Mapping of TEAM-QI components to ITEM**

| **Area of TEAM-QI** | **Where mapped on to ITEM** | **Example / reasoning** |
| --- | --- | --- |
| Questionnaire component (TEAM-U) | Task Design (Features)  Interdependence (2 items), autonomy (2 items), specialised knowledge (2 items), clarity of rules and procedures (6 items), use of quality framework/guidelines (3 items)  15 items* | Items covering team members knowledge of their role and what it involves, having the skills within their team required and the protocols for discussing cases and reviewing data.  Example items:  Use of quality framework/guidelines: In the delivery suite/theatre our team makes good use of structured communication protocols (e.g. checklists, closed-loop communication) |
|  | Team Processes  Communication (3 items), collaboration (3 items), coordination (1 item), conflict (3 items), leadership (6 items), decision making (4 items), participation (6 items)  26 items | Items that cover the skills needed for effective teams.  Example items:  Leadership: Leadership is effective in our team.  Participation: If the patient is not present, someone who has met the patient attends to discuss their case. |
|  | Psychosocial Traits  Cohesion (3 items), norms (2 items), efficacy (5 items), problem-solving effectiveness (2 items)  12 items | Items in the survey that focus on important team qualities needed for effective teamwork.  Example items:  Efficacy: Clinical information is presented to a high standard at our team meetings. |
|  | Organisational Context  Goals/Standards (2 items), Structure/ Characteristics (2 items), Rewards/Supervision (2 items), Resources (3 items), Training environment (4 items), Information system (1 item)  14 items | Items that cover the wider involvement of the organisation or between teams. This includes standards set, inter-team working, the resources both human and technological available.  Example items:  Information system: The organisation provides adequate technology for teamwork (e.g., ability to share information from multiple sources) |
|  | Subjective Outcomes (7 items) | These items cover team members perspectives on their own team. For example, their enjoyment of working in the team and their role, and also their views on the standard of care and recommendations their team provides.  Examples: I feel part of a first-class team, I would like my team to look after a family member or friend if they needed their service. |
| MOT (observation component) | Team Processes  (Communication, collaboration, coordination, conflict, leadership, decision making, participation) | Observations of team meetings using MOT will obtain information on team processes (for example, sections of the MOT cover leadership, teamwork and culture, decision making) |
| Platform | Task Design (type, composition) | E.g., Team information will be entered onto the platform when teams are set up |

** Some items are mapped onto multiple areas of the framework*

**Supplement 7.1 Prototype TEAM-QI core items internal consistency**

| **Core item topic** | **Item-test correlation** | **Item-rest correlation** | **Average item Covariance** | **Alpha** |
| --- | --- | --- | --- | --- |
| Core 1- Team has skills and expertise | 0.11 | 0.07 | 0.21 | 0.92 |
| Core 2- Leadership seeks adequate resource//support | 0.71 | 0.66 | 0.19 | 0.91 |
| Core 3- Effective team leadership | 0.76 | 0.74 | 0.20 | 0.91 |
| Core 4- Leadership are approachable | 0.38 | 0.35 | 0.21 | 0.92 |
| Core 5- Feel value and supported | 0.46 | 0.42 | 0.20 | 0.92 |
| Core 6- Mutual trust and respect | 0.44 | 0.40 | 0.20 | 0.92 |
| Core 7- Clear roles and responsibilities | 0.43 | 0.40 | 0.21 | 0.92 |
| Core 8- Able to contribute to discussions | 0.36 | 0.33 | 0.21 | 0.92 |
| Core 9- No members make teamworking difficult | 0.38 | 0.32 | 0.20 | 0.92 |
| Core 10- Team has teaching and training role | 0.56 | 0.52 | 0.20 | 0.92 |
| Core 11- members training needs supported | 0.68 | 0.65 | 0.20 | 0.91 |
| Core 12- Adequate equipment/technology | 0.39 | 0.33 | 0.20 | 0.92 |
| Core 13- Clear record of team recommendations | 0.63 | 0.59 | 0.20 | 0.92 |
| Core 14- Track patients through the system | 0.65 | 0.62 | 0.20 | 0.91 |
| Core 15- Someone ensures patients know about team | 0.19 | 0.13 | 0.21 | 0.92 |
| Core 16- Patients provided with sufficient information | 0.31 | 0.23 | 0.21 | 0.92 |
| Core 17- Team considers patient views/ preferences/situation | 0.49 | 0.47 | 0.21 | 0.92 |
| Core 18- Like team to care for own friends and family | 0.52 | 0.49 | 0.21 | 0.92 |
| Core 19- Rarely defer decision making due to lack of information | 0.43 | 0.38 | 0.20 | 0.92 |
| Core 20- Organisational support to resolve issues | 0.67 | 0.63 | 0.19 | 0.91 |
| Core 21- Team collect and review data | 0.53 | 0.49 | 0.20 | 0.92 |
| Core 22- Team audits outcomes | 0.40 | 0.36 | 0.20 | 0.92 |
| Core 23- Solve issues as a team | 0.52 | 0.49 | 0.20 | 0.92 |
| Core 24- First-class team | 0.60 | 0.57 | 0.20 | 0.92 |
| Core 25- Organisational support for role | 0.83 | 0.80 | 0.19 | 0.91 |
| Core 26- Teamwork needs improving | 0.02 | -0.03 | 0.21 | 0.92 |
| Core 27- Work well across multiple sites | 0.73 | 0.70 | 0.20 | 0.91 |
| Core 28- Benefit from more face-to-face working | 0.03 | -0.03 | 0.21 | 0.92 |
| Core 29- Leadership effective at managing challenges | 0.78 | 0.76 | 0.20 | 0.91 |
| Core 30- Make evidence-based patient centred recommendations | 0.74 | 0.73 | 0.20 | 0.92 |
| Core 31- Leadership skills | 0.67 | 0.64 | 0.20 | 0.91 |
| Core 32- Rarely blame each other when things go wrong | 0.60 | 0.54 | 0.19 | 0.92 |
| Core 33- Collegiately and cooperation | 0.71 | 0.68 | 0.20 | 0.91 |
| Core 34- Learn from mistakes | 0.63 | 0.62 | 0.21 | 0.92 |
| Core 35- Timely interdisciplinary referrals | 0.58 | 0.54 | 0.20 | 0.92 |
| Core 36- Safe communication between teams | 0.50 | 0.46 | 0.20 | 0.92 |
| Core 37 Team participate in training together | 0.58 | 0.54 | 0.20 | 0.92 |
| Core 38 Training/development is prioritised | 0.60 | 0.56 | 0.20 | 0.92 |
| Core 39 Feel able to speak up | 0.36 | 0.32 | 0.21 | 0.92 |
|  |  |  |  |  |
| **Test scale** |  |  | 0.20 | 0.92 |

**Supplement 7.2 Prototype TEAM-QI meeting items internal consistency**

| **Meeting item topic** | **Item-test correlation** | **Item-rest correlation** | **Average item Covariance** | **Alpha** |
| --- | --- | --- | --- | --- |
| Meeting 1- Attendance of others | 0.48 | 0.42 | 0.24 | 0.87 |
| Meeting 2- Members or deputies present | 0.23 | 0.13 | 0.25 | 0.88 |
| Meeting 3- Quoracy | 0.59 | 0.51 | 0.23 | 0.87 |
| Meeting 4- Clear roles/responsibilities | 0.69 | 0.65 | 0.24 | 0.87 |
| Meeting 5- Make positive contribution | 0.49 | 0.44 | 0.24 | 0.87 |
| Meeting 6- Agreed protocol for meetings | 0.54 | 0.46 | 0.23 | 0.87 |
| Meeting 7- Room/area suitable | 0.71 | 0.63 | 0.21 | 0.86 |
| Meeting 8- Timing convenient | 0.61 | 0.56 | 0.23 | 0.87 |
| Meeting 9- Attendance of self | 0.46 | 0.41 | 0.24 | 0.87 |
| Meeting 10- Comprehensive agenda | 0.60 | 0.52 | 0.22 | 0.87 |
| Meeting 11- Locally agreed minimum datasets | 0.53 | 0.47 | 0.24 | 0.87 |
| Meeting 12- Clear why case/issue is discussed | 0.64 | 0.57 | 0.23 | 0.87 |
| Meeting 13- High standard clinical information | 0.77 | 0.72 | 0.22 | 0.86 |
| Meeting 14- Disruptions/distractions | 0.52 | 0.42 | 0.23 | 0.87 |
| Meeting 15- Clear who to discuss | 0.67 | 0.61 | 0.23 | 0.86 |
| Meeting 16- Someone present knows the patient | 0.26 | 0.17 | 0.24 | 0.88 |
| Meeting 17- When to rediscuss case/issue | 0.56 | 0.49 | 0.23 | 0.87 |
| Meeting 18- Enjoy participating | 0.64 | 0.58 | 0.23 | 0.87 |
| Meeting 19- Adequate privacy | 0.65 | 0.60 | 0.23 | 0.87 |
|  |  |  |  |  |
| **Test scale** |  |  | 0.23 | 0.88 |

**Supplement 7.3 TEAM-QI maternity specific items internal consistency**

| **Maternity items topic** | **Item-test correlation** | **Item-rest correlation** | **Average item Covariance** | **Alpha** |
| --- | --- | --- | --- | --- |
| Maternity 1- Consultant-led handovers high quality | 0.73 | 0.55 | 0.28 | 0.67 |
| Maternity 2- Working with other teams | 0.55 | 0.34 | 0.33 | 0.68 |
| Maternity 3- Listening to women/families | 0.54 | 0.22 | 0.34 | 0.71 |
| Maternity 4- Managing high risk/complex cases | 0.73 | 0.23 | 0.32 | 0.79 |
| Maternity 5- Good use of structure protocols | 0.80 | 0.68 | 0.24 | 0.62 |
| Maternity 6- Situational awareness | 0.66 | 0.54 | 0.32 | 0.68 |
|  |  |  |  |  |
| **Test scale** |  |  | 0.30 | 0.73 |

**Supplementary Table 8: Summary of free text feedback on TEAM-U (n=16)**

| **Main theme** | **Sub-theme (Number of team members supporting theme)**  ***Mapped to CFIR domain*** | **Illustrative quotes** |
| --- | --- | --- |
| Strengths | The value in being able to reflect and input as a team, hear everyone’s voice (9)  *Intervention characteristics: strengths and qualities* | “has made me think about how the team works”  “Collectively there may be better ideas and not a narrow view”  “Without the opinions of team members and how they perceive the working of the team then positive changes cannot be made” |
|  | Comprehensive content (3)  *Intervention characteristics: strengths and qualities* | “Covers a good range of aspects, useful to see where we all stand and if there is consensus”  “All questions easy to understand and relevant to my role” |
| Possible limitations | Specificity and appropriateness (4)  *Intervention characteristics: strengths and qualities* | “The bit about working in acute environments - handover/ theatre wasn't really relevant to this team meeting”  “the question needs to qualify which team this refers to as we work in multiple teams” |
|  | Effectiveness based on how the data is used (3)  *Readiness for Implementation* | “It depends how the feedback from the survey is utilised”  “I wonder what the response rate will be so how representative the feedback will be” |
|  | Wording and rating of items (2)  *Intervention characteristics: strengths and qualities* | Some of the questions were a bit technical! Less jargon might have made it easier to fill in |
